# A case report of interstitial pneumonia induced by vedolizumab in a patient with ulcerative colitis

**DOI:** 10.1097/MD.0000000000039195

**Published:** 2024-08-02

**Authors:** Ailing Liu, Susu Ye, Zeyuan Diao, Hua Liu, Yonghong Xu, Jun Wu, Tao Mao, Zibin Tian, Xueli Ding

**Affiliations:** a Department of Gastroenterology, the Affiliated Hospital of Qingdao University, Qingdao, China; b Liver Disease Center, The Affiliated Hospital of Qingdao University, Qingdao, China.

**Keywords:** interstitial pneumonia, ulcerative colitis, vedolizumab

## Abstract

**Rationale:**

The interstitial pneumonia (IP) linked to vedolizumab (VDZ) in patients with ulcerative colitis (UC) is rare. Prompt diagnosis and treatment can improve patient outcomes.

**Patient concerns:**

A 39-year-old man with UC who received VDZ as sole therapy developed symptoms such as chest tightness, cough, and suffocation.

**Diagnoses:**

IP was confirmed through pulmonary function tests, chest computed tomography, and bronchoscopic biopsy.

**Interventions:**

The patient was given methylprednisolone and VDZ cessation.

**Outcomes:**

The patient’s symptoms improved and remained symptom-free after nearly 2 years.

**Lessons:**

VDZ-induced IP should be considered when evaluating pulmonary infections in UC patients treated with VDZ.

## 1. Introduction

Vedolizumab (VDZ), a humanized lgG monoclonal antibody targeting integrin α4β7, selectively acts in the intestine. It effectively induces mucosal healing and clinical remission in ulcerative colitis (UC) patients. Despite its efficacy, adverse reactions, such as kidney and liver dysfunction, joint pain, gastrointestinal discomfort, rash, and infusion reactions, have been reported. Although most are mild and transient,^[1]^ some require drug withdrawal due to severity. Rare reports exist of VDZ causing interstitial pneumonia (IP).^[2–5]^ We present China’s first reported case of a UC patient developing IP during VDZ therapy.

## 2. Case report

A 39-year-old man with a 3-year history of UC was hospitalized on July 28, 2022, due to a 3-week chest tightness, cough, and suffocation. UC was diagnosed after symptoms of mucopurulent bloody stool. Initially treated with sulfasalazine, he later switched to prednisone and mesalazine due to fever and rash. He had been in remission with VDZ for over a year. The new cough, with yellow-white sputum, worsened with exertion but improved with rest. He denied fatigue, fever, hemoptysis, or night sweats but reported an ineffective course of azithromycin and a 5 kg weight loss. Wet and dry rales were heard in both lungs on examination.

**Figure 1.**
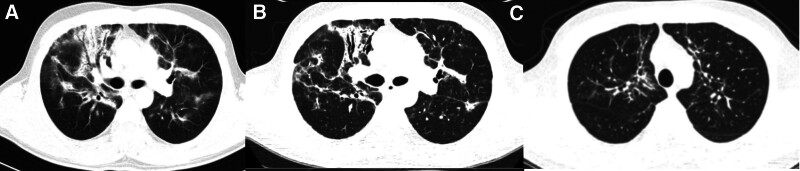
Chest CT scans showing the progression and resolution of findings over time. (A) July 2022: Bilateral ground-glass areas and interstitial opacities. (B) August 2022: Resolution of bilateral interstitial opacities and lobar consolidation. (C) February 2024: Development of multiple pulmonary nodules. CT = computed tomography.

Laboratory findings indicated low albumin levels (33.24 g/L), an erythrocyte sedimentation rate of 28 mm/h, and a C-reactive protein (CRP) level of 22.72 mg/L. Blood gases showed low oxygen levels (pH 7.35, partial pressure of carbon dioxide 45 mm Hg, oxygen partial pressure 66 mm Hg). Tumor markers (carcinoma embryonic antigen, α-fetoprotein, and serum carbohydrate antigen 199) were negative, while antinuclear antibody and anti-neutrophil cytoplasmic antibodies perinuclear were positive. Routine blood and renal function tests, procalcitonin levels, and blood bacterial cultures were negative. Sputum cultures for bacteria, mycobacteria, and fungi were also negative. Cardiac, thyroid, and abdominal ultrasound findings were unremarkable. Pulmonary function tests revealed severe diffusion dysfunction and moderate restrictive ventilatory dysfunction. Chest enhanced computed tomography (CT) scan (Fig. 1A) showed bilateral ground-glass areas, interstitial opacities, and lobar consolidation primarily in the middle zone of the upper lung, along with unobstructed bronchiectasis, indicating IP. Bronchoscopy revealed soft tissue in the right upper lung’s anterior segment. Histopathology indicated chronic inflammation, interstitial fibrous tissue growth, histiocyte aggregation in alveolar cavities, plasma and lymphocyte cell infiltration, alveolar epithelial hyperplasia, and fibroblast cluster formation, with no malignant cells detected.

After excluding pulmonary bacterial, tuberculosis and fungal infections through comprehensive examinations, the patient received a diagnosis of VDZ-associated IP. VDZ therapy was stopped, and intravenous methylprednisolone 40 mg qd was initiated, leading to improvement in symptoms such as expectoration, cough, shortness of breath, and chest tightness, as confirmed by chest enhanced CT scan (Fig. 1B). He was discharged on August 26, 2022.

During the February 2024 follow-up, prednisone was gradually reduced to 8 mg qd for maintenance, and mesalazine 1 g qid po was started. The patient remained symptom-free with one daily bowel movement and gained 3 kg. Blood tests for routine markers, erythrocyte sedimentation rate, and CRP were normal. The chest-enhanced CT scan also indicated improvement (Fig. 1C).

## 3. Discussion

Interstitial lung diseases often involve inflammation or fibrosis in the interstitial space and can be caused by factors like long-term dust inhalation, medication use, or autoimmune diseases.^[6,7]^ Symptoms commonly include breathlessness and reduced exercise tolerance. Tests typically show restrictive ventilatory dysfunction, decreased diffusion capacity, and hypoxemia. Imaging may reveal reticular shadows, ground-glass opacities, cysts, honeycombing, consolidation, and abnormal linear shadows. Pathological examination may show alveolitis, diffuse pulmonary consolidation, and interstitial fibrosis. Treatment often includes glucocorticoids. In this case, the patient had been symptom-free while using VDZ long-term but later developed symptoms such as chest tightness, cough, and shortness of breath. Imaging, pulmonary function tests, and bronchoscopic biopsy indicated IP. Discontinuation of VDZ and treatment with prednisolone proved effective, confirming the diagnosis of VDZ-related IP.

Pugliese et al studied 10 inflammatory bowel disease patients who developed IP during VDZ therapy.^[2]^ Patients had received a median of 4 VDZ infusions over 12 weeks before IP onset. Although inflammatory bowel disease was quiescent or mild, CRP levels increased. Common symptoms were fever, cough, and dyspnea. Chest X-rays revealed peribronchial thickening, bilateral interstitial opacities, multiple pulmonary nodules, and lobar consolidation. Respiratory virus serology and sputum cultures were negative. One patient’s pulmonary biopsy showed macrophage desquamation and chronic mononuclear interstitial infiltrates with obliterative and eosinophilic aspects. Pulmonary function tests in 2 patients showed mild to moderate restrictive ventilatory patterns with reduced carbon monoxide diffusion. Antibiotic therapy (oral fluoroquinolones or macrolides and/or parenteral penicillins) failed. All patients received corticosteroids (prednisolone equivalent dose, 50 mg/d, tapered over 6 weeks), resulting in prompt symptom and radiographic improvement (median follow-up, 7 weeks). One patient resumed VDZ after pneumonitis resolved but relapsed after 2 weeks, requiring VDZ discontinuation and a new course of oral corticosteroids, leading to improvement.

The specific mechanism behind VDZ-related IP remains uncertain.^[2,5]^ VDZ’s interaction with α4β7 causes it to be taken up by cells, leading to increased presence of other integrins on the surface of white blood cells. This change may redirect these inflammatory cells from the gut to other areas, where they can cause immune-mediated damage. Lung endothelial cells react to antigen presentation by increasing the expression of E- and P-selectin, which bind to α4β1 integrin. VDZ therapy boosts the expression of α4β1 integrin on gut white blood cells, potentially initiating an inflammatory response through the activation of inflammatory mediators, cytokines, and angiogenic factors.

## 4. Conclusions

Here we report the first case of VDZ-induced interstitial IP in a patient with UC in China. When VDZ-treated patients with UC experience cough and shortness of breath, drug-induced IP should be considered, along with the possibility of pulmonary infection. The limitation of our article include limited availability of broncho-alveolar lavage that could exclude mycobacterial and viral infection. Prompt chest CT scans, pulmonary function tests, and bronchoscopic biopsies can confirm the diagnosis, guide treatment, and enhance patient outcomes.

## Author contributions

**Writing – original draft:** Ailing Liu, Susu Ye, Zeyuan Diao.

**Investigation:** Hua Liu, Yonghong Xu, Jun Wu.

**Writing – review & editing:** Tao Mao, Zibin Tian, Xueli Ding.
